# Preferred orientation and its effects on intensity-correlation measurements

**DOI:** 10.1107/S2052252521012422

**Published:** 2022-01-21

**Authors:** Jack Binns, Connie Darmanin, Cameron M. Kewish, Sachini Kadaoluwa Pathirannahalge, Peter Berntsen, Patrick L. R. Adams, Stefan Paporakis, Daniel Wells, Francisco Gian Roque, Brian Abbey, Gary Bryant, Charlotte E. Conn, Stephen T. Mudie, Adrian M. Hawley, Timothy M. Ryan, Tamar L. Greaves, Andrew V. Martin

**Affiliations:** a School of Science, RMIT University, Melbourne, Victoria 3000, Australia; b ARC Centre of Excellence in Advanced Molecular Imaging, La Trobe Institute for Molecular Sciences, La Trobe University, Victoria 3086, Australia; c Australian Nuclear Science and Technology Organisation, Australian Synchrotron, Victoria 3168, Australia; d Department of Chemistry and Physics, La Trobe Institute for Molecular Science, La Trobe University, Victoria 3086, Australia

**Keywords:** correlated fluctuations, dynamical studies, XFELs, X-ray free-electron lasers, fluctuation scattering, preferred orientation, pair-angle distribution functions, intensity correlations, crystalline domains

## Abstract

Intensity-correlation measurements allow access to nanostructural information on a range of ordered and disordered materials beyond traditional pair-correlation methods. In real space, this information can be expressed in terms of a pair-angle distribution function (PADF) which encodes three- and four-body distances and angles. To date, correlation-based techniques have not been applied to the analysis of microstructural effects, such as preferred orientation, which are typically investigated by texture analysis. Preferred orientation is regarded as a potential source of error in intensity-correlation experiments and complicates interpretation of the results. Here, the theory of preferred orientation in intensity-correlation techniques is developed, connecting it to the established theory of texture analysis. The preferred-orientation effect is found to scale with the number of crystalline domains in the beam, surpassing the nanostructural signal when the number of domains becomes large. Experimental demonstrations are presented of the orientation-dominant and nanostructure-dominant cases using PADF analysis. The results show that even minor deviations from uniform orientation produce the strongest angular correlation signals when the number of crystalline domains in the beam is large.

## Abstract 2

The theory of preferred orientation in fluctuation scattering is developed and demonstrated in experimental studies of self-assembled lipid materials.

## 1. Introduction

Many functional materials exhibit polycrystalline microstructures. The physical properties of these materials depend both on the structure of each single crystal domain as well as the spatial orientation and distribution of these domains – the texture – of the aggregate (Bunge, 1982 ▸, 1987 ▸; Engler & Randle, 2010 ▸). Many technologically important physical properties such as bulk moduli, piezoelectric coefficients, ionic conductivity and superconductivity arise due to anisotropy in the crystal structure, and the resulting macroscopic behaviour of a polycrystalline material is governed by its texture (Fuentes, 1998 ▸; Welzel *et al.*, 2005 ▸; Hilgenkamp & Mannhart, 2002 ▸). Anisotropy in the orientation distribution, known as preferred orientation, has significant consequences for bulk physical behaviour and complicates the analysis of diffraction data by modifying peak intensities. In powder-diffraction studies concerned primarily with structure determination, texture frequently complicates the analysis, but modern refinement software contains routines for correcting intensity modulations following well known corrections (*e.g.* Dollase, 1986 ▸; Ahtee *et al.*, 1989 ▸; Järvinen, 1993 ▸; Zolotoyabko, 2009 ▸).

Texture theory for polycrystalline materials is well developed and has been widely applied in particular to metals and ceramics (Bunge, 1982 ▸). In this formalism, the texture of a sample is described by the orientation distribution function, which is experimentally accessible through its two-dimensional projection, the pole distribution. A distinction can be made between macrotexture and microtexture (Engler & Randle, 2010 ▸). Macrotexture occurs when the preferred orientation of crystal grains is correlated to the macroscopic dimensions of the sample. This type of texture is accessible to conventional X-ray and neutron scattering methods. Microtexture occurs when there are microscopic regions in the sample where crystal grains align locally, and can be studied using techniques such as electron backscatter diffraction (Dingley & Randle, 1992 ▸; Wright *et al.*, 2007 ▸; Engler & Randle, 2010 ▸).

The importance of texture is not limited to crystalline materials. Many composites such as biological tissues, fabrics and carbon fibre gain their emergent properties from the orientation and hierarchical organization of fibres, domains or nanoparticles (Aghamohammadzadeh *et al.*, 2004 ▸; Kelly *et al.*, 2018 ▸; Cho *et al.*, 2017 ▸; Qian *et al.*, 2018 ▸; Poulsen *et al.*, 2005 ▸).

Methods for probing disordered or partially ordered structures are less well developed than classical crystallography. Typically, disordered materials have been studied through the analysis of the ‘total scattering structure factor’ and its Fourier transform, the pair distribution function (PDF). This highly successful approach has been applied to a wide array of materials taking advantage of advances in instrumentation and modelling techniques, including development of texture analysis (Paddison, 2019 ▸; Dippel *et al.*, 2019 ▸; Cervellino & Frison, 2020 ▸). Fluctuation X-ray scattering (FXS) is an alternative approach for probing disordered structure (Kam, 1977 ▸; Kam *et al.*, 1981 ▸; Treacy *et al.*, 2005 ▸) that aims to go beyond pair statistics and recover information about local angular structure. By angular correlation analysis of large ensembles of X-ray scattering data, FXS is able to extract atomic and nanoscale structural information from a range of materials using both synchrotron and X-ray free-electron laser sources, including colloidal glasses and crystals (Wochner *et al.*, 2009 ▸; Lehmkühler *et al.*, 2016 ▸), liquid-crystal membranes (Kurta *et al.*, 2013 ▸; Zaluzhnyy *et al.*, 2015 ▸, 2018 ▸), nanoparticles and viruses (Mendez *et al.*, 2014 ▸, 2016 ▸; Kurta *et al.*, 2017 ▸, 2019 ▸; Pande *et al.*, 2018 ▸), and magnetic domains (Su *et al.*, 2011 ▸).

Recently we demonstrated how averaged angular correlations from diffraction patterns can be transformed into a three-dimensional multi-atom real-space distribution called the pair-angle distribution function (PADF) (Martin, 2017 ▸; Martin *et al.*, 2020*a* ▸,*b* ▸; Adams *et al.*, 2020 ▸). The PADF is a higher-order analogue of the basic PDF and is rich in information relating to orientation and bond angles. In the PADF, this information is mapped into a sum of three- and four-atom correlation functions, which encode two pairwise distances and one relative angle.

One underlying assumption in PADF analysis and many other three-dimensional FXS analysis methods is a uniform orientation distribution of scatterers. For fluctuation scattering and PADF analysis to be more widely adopted, texture and its effect on the PADF must be explored. Here we develop theory to quantify the contribution arising from the preferred orientation in FXS and compare it with the desired nanostructure FXS signal. We consider a limiting case of microtexture, where the orientation distribution is close to, but not perfectly, uniform. Since FXS experiments typically aim for materials with a uniform orientation distribution, this microtextural case is the most likely way that preferred orientation will affect FXS experiments. In this case, we find that the preferred-orientation effects dominate when the number of domains (or particles) in the beam becomes very large. We also present a real example observed in a self-assembled lipid system where preferred orientation is the dominant effect observed in the PADF/FXS results. Subsequently, we compare this with a microfocus FXS experiment which circumvents preferred orientation and where the desired nanostructure signal is dominant.

## 2. Theory

### 2.1. Preferred orientation

Texture analysis is concerned with the probability distribution of crystallite orientations in polycrystalline materials. We define this probability as *f*(*g*), where *g* represents a rotation of a crystallite from a sample orientation axis. The rotation can be represented, for example, in terms of Euler angles: *g* = {ϕ_1_, Φ, ϕ_2_}. Explicitly, *g* relates the rotation of a crystallite-fixed coordinate system (**x**, **y**, **z**) to a sample orientation coordinate system 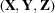 . Here we follow the spherical harmonic formalism, first presented by Roe (1965 ▸), using notation given by Bunge (1982 ▸). In spherical harmonics, the orientation distribution is written as

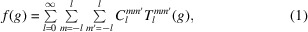

and if the orientation *g* is expressed in terms of Euler angles we obtain 

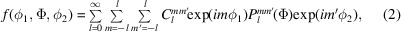

where 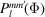 are generalizations of the associated Legendre functions. When the crystal domains have no preferred orientation, *f*(ϕ_1_, Φ, ϕ_2_) takes a constant value independent of crystal orientation. Crystal point-group symmetry can act to make certain rotations symmetrically equivalent and also places constraints on the coefficients 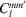 (Bunge, 1982 ▸; Von Dreele, 1997 ▸). However, our treatment will remain general and applicable to crystalline and amorphous materials. The orientation distribution is not directly measurable. However, X-ray diffraction experiments can measure a reduced form of *f*(ϕ_1_, Φ, ϕ_2_) called the pole distribution (or ‘general axis distribution’), which is expressed with respect to a sample orientation axis **y** as 

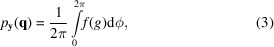

where ϕ is an angle of the polar axis of a crystallite **y** to the scattering vector **q** [see Roe (1965 ▸), Bunge (1982 ▸) and Paakkari *et al.* (1988 ▸) for further geometrical details]. In spherical harmonics, the pole distribution is given by 

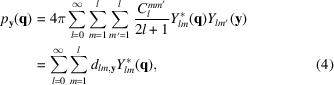

where the coefficients 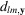 are defined by 

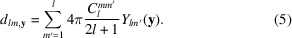

In the case where the orientation distribution is not uniform, the pole distribution 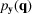 expresses this anisotropy and is related to the measured diffraction pattern 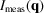 by 

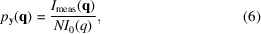

where *I*_0_(*q*) is the isotropic intensity distribution of a single domain and *N* is the number of particles per diffraction pattern. Although we write 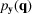 to be consistent with the right-hand side of equation (6), the pole distribution depends on orientation only and is independent of the magnitude of the scattering vector *q*.

In this study, only equations (4) and (6) are needed. Equation (6) will be used to write down the correlation function in terms of the pole distribution, while the spherical harmonic coefficients 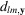 introduced in equation (4) will be used to estimate the size of the preferred orientation signal in intensity-correlation analysis.

### 2.2. Intensity correlations

There are two types of texture that we classify here as macrotexture and microtexture. In the case of macrotexture, the distribution of the 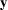 axis for each crystal domain is aligned to a macroscopic dimension of the sample. The orientation can be chosen with respect to the beam axis and, possibly, rotated to recover three-dimensional information about the pole distribution. In the case of microtexture, the axis 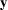 for each crystal domain is associated with a region of the sample larger than a single crystal domain but much smaller than the macroscopic dimensions of the sample. Within each region, a crystal domain has a preferred orientation with respect to a local value of 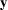 , but not necessarily to the macroscopic dimensions. The experimental data we present are an example of microtexture but not macrotexture.

In a fluctuation-scattering experiment, we measure a large ensemble of diffraction patterns from different microscopic regions of the sample by raster scanning. For each measurement labelled *k*, we can specify a reference axis 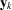 . The orientation distribution *f*_*k*_(*g*) is then specified with respect to this local reference axis. Then we define 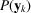 to be the distribution of directions 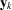 in the ensemble of measured regions of the sample. In the case of macrotexture, we expect 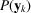 to be sharply peaked around a particular direction related to the macroscopic dimensions of the sample. For microtexture, 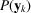 can be broad or even uniform and set by microstructural properties such as local strain fields.

We denote the measured diffraction pattern for each region *k* as 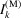 . We define the intensity with the angular mean subtracted by 

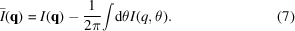

The measured correlation function is given by 

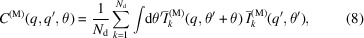

where *N*_d_ is the number of measured diffraction patterns.

Our aim here is to predict the expectation value (ensemble average) of *C* ^(M)^(*q*, *q*′, θ) from the orientation distribution *f*(*g*). For notational simplicity, we denote the expectation value of the correlation function by *C*(*q*, *q*′, θ). We denote the diffraction pattern of a crystal in region *k* and in orientation *g* by *I*_*k*_(**q**; *g*). For our purposes here, it is sufficient to associate *I*_*k*_(**q**; *g*) to a single crystal structure throughout the sample. Structural heterogeneity can be included by considering *I*_*k*_(**q**; *g*) as an ensemble average over the different structures or unit cells present. The rotation *g* is defined with respect to the reference axis for that region 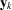 . The expected value of the correlation function is then 

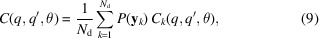

where 

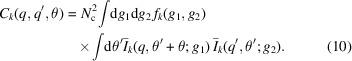

Here we have introduced a joint orientation distribution *f*(*g*_1_, *g*_2_) that describes the joint probability of finding two crystals in orientations *g*_1_ and *g*_2_ in the same microscopic region of the sample.

Equations (9) and (10) ignore the coherent interference between nearby crystal domains. This is not generally true but is a suitable approximation when the coherence length of the beam is smaller than the crystal domain. Even when the coherence length exceeds the crystal-domain size, these interference effects will appear only as modulations in the vicinity of a Bragg peak or a powder ring. In correlation analysis, this manifests as fine details on the angular correlations generated by the unit cell. In PADF analysis, we will set a maximum real-space distance to be of the order of the unit-cell size, much smaller than the inter-domain separation distance. Hence PADF analysis sets an effective coherence length that ignores these coherence effects.

In the case where *g*_1_ = *g*_2_, the diffraction of a single crystal correlates with itself and we have *f*_*k*_(*g*_1_, *g*_2_) = *f*_*k*_(*g*_1_)δ(*g*_2_ − *g*_1_)/*N*_c_, where *N*_c_ is the average number of crystals illuminated per exposure. Here the delta function is used as a convenient notation and is understood as a distribution that always appears within the integral given by equation (10). In the case where *g*_1_ ≠ *g*_2_, we can make a simplification that these two crystals independently sample the orientation distribution, so that *f*_*k*_(*g*_1_, *g*_2_) ≃ *f*_*k*_(*g*_1_)*f*_*k*_(*g*_2_). Making these simplifications we can write the correlation function in two parts: 

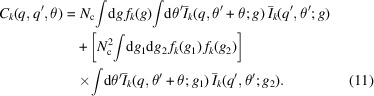

The first term on the right-hand side of equation (11) (top line) contains the nanostructural term. Many FXS analysis methods, including PADFs, assume that the orientation distribution *f*_*k*_(*g*) is uniform. This assumption breaks in the case of preferred orientation, *i.e.* when *f*_*k*_(*g*) is not uniform.

The second term (second and third lines) correlates the diffraction from different crystals and averages to zero if the orientation distribution is uniform. Hence, it is neglected in many FXS analysis methods. If there is a preferred orientation, the second term will not be zero and it will contribute to the FXS correlation signal.

### 2.3. Texture contribution

Texture effects arise due to the presence of many domains in the exposure. To give the trivial extreme, when the number of domains is large and their orientational distribution is uniform, we enter the regime of ideal powder diffraction. In this instance, correlation functions are uniform in θ so there is no angular intensity variation.

In the limit such that the number of domains is large and they exhibit a non-uniform orientation distribution, we can write 

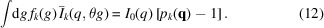

The interparticle correlation term [latter term in equation (11), lines two and three] is approximated as 

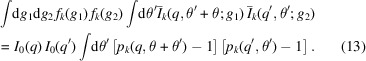

In the following subsection, we will consider the interparticle term in the case of microtextured samples.

### 2.4. A special case for microtexture

Let us consider a special case of microtexture, which will prove useful for analysing the experimental case we present in our results. In particular, the case where the sample is untextured on a macroscopic scale and in the limit where the crystal orientation distribution 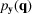 is almost uniform. Consequently, we assume that local regions with orientations 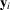 have a uniform distribution, so that 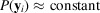 . Secondly, we assume that the crystal orientation distribution is very close to uniform, such that 

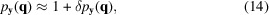

where 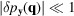 . In this special case the correlation function simplifies to the form 

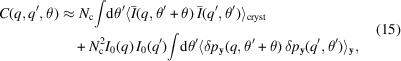

where *N*_c_ is the average number of crystals illuminated per measurement, 〈〉_cryst_ is the average contribution per crystal and 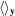 is the average contribution per region.

For this case, the nanostructural term is not affected by the preferred orientation and scales with the number of particles illuminated per exposure *N*_c_. Preferred orientation arises through the second term *via* the correlation between different crystallites in the same microstructural region, which scales with 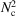 . Hence, even though we have assumed an almost uniform distribution ( 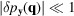 ), the preferred-orientation effect could dominate if *N*_c_ is very large.

### 2.5. Comparison of nanostructure and texture terms

To further quantify the relative strength of the nanostructure and texture terms, we now consider spherical harmonic analysis of the intensity-correlation function (Kam, 1977 ▸; Kirian, 2012 ▸; Kurta *et al.*, 2016 ▸).

We can use the spherical harmonics to define total scattering power in the angular part of the spectrum for both the nanostructure and the preferred-orientation terms. The angular power for the nanostructural term is given by 

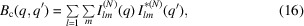

where 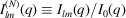 , and *I*_*lm*_(*q*) are the spherical harmonic coefficients of the crystal diffraction. For the preferred-orientation term we have 

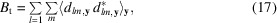

where 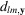 are spherical harmonic coefficients of the pole distribution defined in equation (4).

The terms *B*_c_ and *B*_t_ are related to the harmonic coefficient matrices *B*_*l*_(*q*, *q*′), which are directly obtained from the correlation function. Extracting these matrices is a key step en route to three-dimensional imaging of single particles *via* correlation analysis (Saldin *et al.*, 2010 ▸, 2011 ▸; Liu *et al.*, 2013 ▸; Pande *et al.*, 2014 ▸, 2018 ▸; Donatelli *et al.*, 2015 ▸; Kurta *et al.*, 2017 ▸). It is also a key step in generating the PADF (Martin, 2017 ▸). Here the *B*_*l*_(*q*, *q*′) matrices provide a useful way to quantify the relative strength of the nanostructure and preferred-orientation effects. For the correlation function given by equation (15), the *B*_*l*_(*q*, *q*′) matrices have two contributions: 

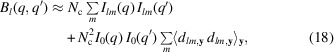

where **y** is now a single sample orientation specified by a choice of coordinate axes.

The relative balance of the terms in equation (18) indicates whether texture or nanostructural effects will come to dominate the PADF: 

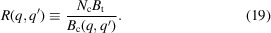

**Figure 1:**
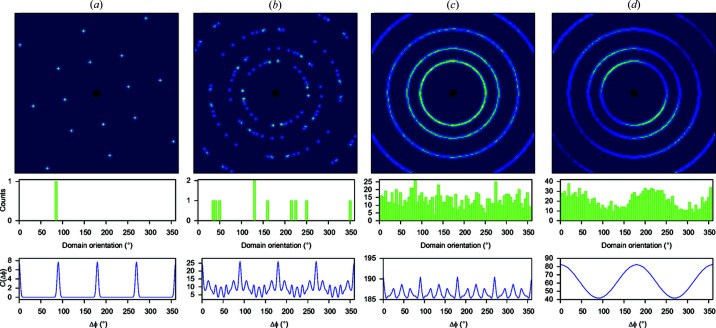
A schematic showing varied X-ray scattering regimes illustrating the effect of crystallite number and pole distribution in each of the following cases: (*a*) a perfect single crystal; (*b*) a small number (*N*_c_ = 10) of domains with uniform pole distribution; (*c*) a large number (*N*_c_ = 1000) of domains, again with uniform pole distribution; and (*d*) *N*_c_ = 1000 with anisotropic pole distribution. (Bottom) Histograms indicating the domain orientations and angular correlation functions [*C*(Δϕ)] calculated for the first diffraction ring for the different types of scattering regimes.

When 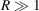 , the preferred-orientation terms dominate over the nanostructural signal. When 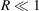 , the nanostructural terms dominate. The number of crystalline domains in the beam *N*_c_ determines which regime is probed in an experiment, which can be controlled with the beam size. Reducing the beam size will reduce *N*_c_ and increase the nanostructural contribution [as shown in Figs. 1 ▸(*a*) and 1 ▸(*b*)]. In this regime, the PADF can no longer be interpreted in terms of three- and four-body arrangements. Conversely, increasing *N*_c_, *i.e.* increasing beam size, will reduce the nanostructure contribution and at the limit wash out all angular correlations [Fig. 1 ▸(*c*)]. In this case, even if the orientation distribution is only weakly peaked, a large number of domains per exposure will cause this term to dominate [Fig. 1 ▸(*d*)].

In the following sections, we describe FXS–PADF experiments conducted on a hexagonal self-assembled lipid phase. When using a relatively large incident beam, we observe a dominant preferred-orientation effect and explore the underlying microtextural information in the PADF. In subsequent experiments using a microfocussed beam, we circumvent preferred orientation to examine nanostructural features directly, demonstrating the importance of beam choice in FXS experiments.

## 3. Methods

### 3.1. Sample preparation and small-angle X-ray scattering

#### 3.1.1. SAXS–WAXS experiments

Cetyltrimethylammonium bromide (CTAB) was mixed with water at 50 mol% concentration to create a hexagonal mesophase (Auvray *et al.*, 1989 ▸). This material was injected into 5 × 5 mm windows in a steel holder and sealed between Kapton polyamide tape. The estimated sample chamber volume of each window is 15 µl. The holder was inserted into a heating chamber which consisted of two large Kapton windows on either side of the steel holder. Once sealed, the heating chamber was connected to a heating bath whose temperature was adjusted so that hot air was pumped into the chamber, increasing the overall temperature of the samples. The chamber temperature was allowed to equilibrate for *ca.* 30 min before data collection. Data were collected between 30 and 48.5°C. One sample chamber contained a thermocouple immersed in water to provide temperature measurement.

SAXS data were collected at the SAXS/WAXS (small-angle/wide-angle X-ray scattering) beamline at the Australian Synchrotron using a Pilatus 1M detector with 12.81 keV X-ray photons (λ = 1.033 Å), and the beam size was 25 × 250 µm. The transmitted flux was 4 × 10^12^ photons s^−1^. Each dataset consisted of 1000 patterns. Reduced (one dimensional) radial plots were generated with *scatterBrain* (Australian Synchrotron, 2019 ▸).

#### 3.1.2. XFM experiments

CTAB was manually mixed with water at compositions of 25, 30, 35 and 40 mol% to study the composition-driven transition from micellar to hexagonal phases (Auvray *et al.*, 1989 ▸). Samples were dispensed into the wells of Greiner 96-well microplates, then heated above the Krafft temperature and vortexed to ensure efficient mixing. The estimated sample volume in each well was 100 µl. Samples were maintained at a hutch temperature of 26°C. The samples were mounted on a plate holder, and data were collected by rastering across each well.

At the X-ray fluorescence microscopy (XFM) beamline, scattering data were collected on an EIGER2 X 1M (Dectris AG, Baden-Daettwil, Switzerland) with 18.5 keV X-rays (λ = 0.6702 Å). The beam size was 2 × 2 µm. The samples were each scanned over a 1 × 1 mm area in 10 µm steps producing 10 000 diffraction patterns of 0.1 s exposure time each, per dataset. Beam intensity was 1.6 × 10^10^ photons s^−1^. The beam centre and detector distance were calibrated to 0.694 m using the first diffraction ring of silver behanate (AgC_22_H_43_O_2_, *d*_001_ = 58.380 Å) using a custom Python script.

### 3.2. Ensemble filtering and orientation analysis

**Figure 2:**
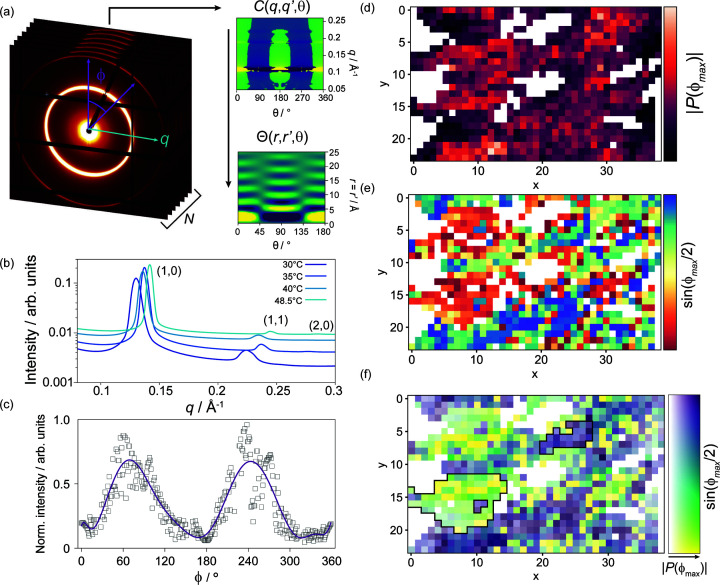
(*a*) From an ensemble of SAXS patterns, a three-dimensional *q*-space intensity-correlation function is generated. This correlation function is transformed into a three-dimensional real-space PADF. In this ensemble, we show a representative SAXS pattern from the hexagonal phase of CTAB–H_2_O. Intensity modulations around the ring (as a function of ϕ) are weak and the smooth rings indicate the presence of many domains within the beam. (*b*) Standard SAXS patterns confirm the hexagonal mesophase structure. (*c*) Close inspection reveals a preferred-orientation effect with characteristic twofold angular peaks. Determination of the (*d*) magnitude [*P*(ϕ_max_)] and (*e*) angle (ϕ_max_) of the orientation combined to (*f*) indicate the presence of macroscopic domains with shared orientations. White pixels indicate data points omitted during the filtering stage. Black lines illustrate examples of domains in this sample.

In the SAXS analysis, two-dimensional image data were integrated to produce reduced one-dimensional intensity *versus q* profiles. Visual observation of the sample chambers indicated the presence of inhomogeneities such as air bubbles or phase changes due to localized deviation in water content. Such inhomogeneities arise intrinsically due to the sample preparation and loading process, so we developed a series of Python scripts to filter and sort the large numbers of SAXS patterns. These data were filtered on the basis of peak positions and relative ratios (1: 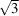 : 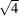 for the hexagonal mesophase) to select single-phase data with unit-cell dimensions within 5% of the ensemble average (Fig. 2 ▸).

Data collected on the SAXS/WAXS beamline displayed preferred orientation on visual inspection, as indicated by the twofold intensity maxima around the strong first diffraction peak. To analyse azimuthal intensity distributions, image-plate data were cropped and rebinned, and the intensities as a function of azimuthal angle ϕ were determined around the most intense first (10) diffraction peak. The resulting intensity *versus * ϕ data were interpolated with a sixth-order polynomial to identify the magnitude and peak angle of any orientational distribution using a Python script (Harris *et al.*, 2020 ▸).

Data collected from the XFM beamline (Howard *et al.*, 2020 ▸) were filtered for outliers using the following process: for each dataset an average one-dimensional intensity *versus q* profile was determined. Each diffraction pattern *k* was reduced, and a profile agreement factor 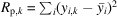 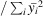 was calculated and used as a figure of merit to select homogeneous subsets of each run.

### 3.3. Pair-angle distribution functions and simulated data

Real-space PADFs were calculated following the work of Martin (2017 ▸). The PADF is obtained by calculating an angular intensity-correlation function from each ensemble of SAXS patterns (Kirian, 2012 ▸). Explicitly, the PADF is a three-dimensional distribution of local two-, three- and four-atom arrangements, and takes the form 

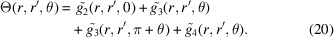

Here 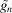 are *n*-body correlation functions, *r* and *r*′ are the interatomic distances of two pairs of atoms (which may share a common atom), and θ is the relative angle between the two atom pairs.

The three-dimensional *q*-space correlation functions required for PADF analysis are given by 

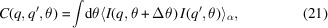

where *I*(*q*′, θ) is a two-dimensional diffraction pattern expressed in polar coordinates (*q*, θ) and 〈〉_α_ is an average over an ensemble of measurements. The angular correlation functions are converted into the PADF using the procedure outlined in detail by Martin (2017 ▸). Briefly, the scattered intensity is represented in a spherical harmonic basis, 

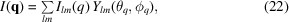

then information about *I*_*lm*_(*q*) can be extracted from the correlation function using 

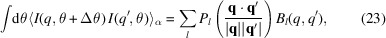

where 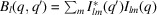 was recovered by inverting equation (23) with singular value decomposition. The matrices are transformed into real space by numerically applying the spherical Bessel transform for both *q* and *q*′ variables at each value of *l*, 

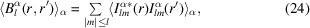

and summing the resulting real-space functions weighted by the Legendre polynomials: 

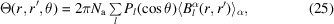

where *N*_a_ is the number of atoms in the sample.

Simulated SAXS datasets were generated using the averaged *q*-space positions and intensities of the corresponding experimental patterns. To simulate the effects of preferred orientation, the uniform radial intensity was modulated with a twofold sinusoidal term or Lorentzian peak with a randomized peak angle (ϕ_max_) to simulate the microtexture observed experimentally. These simulated images were treated with the same processing pipeline as the corresponding cropped and rebinned experimental patterns.

## 4. Observations and simulations

### 4.1. Scattering from microtextured samples

Initial analysis of the SAXS patterns indicated the formation of an *H*_*I*_ hexagonal lipid phase as expected for the 50 mol% CTAB–H_2_O mixtures at room temperature (Auvray *et al.*, 1989 ▸). Phase identification was based on the characteristic ratios of the (10) and (11) peaks. The (20) peak can be observed at higher temperatures [Fig. 2 ▸(*b*)], although as noted elsewhere, the intensities of these higher-order peaks are very low for *H*_*I*_ CTAB–H_2_O, possibly due to surface roughness of the water/lipid cylinders or simply due to the form-factor intensity for CTAB–H_2_O at these *q* values (Seddon, 1990 ▸; Yang & White, 2006 ▸). Examination of the diffraction rings showed the presence of twofold radial intensity maxima in many of the patterns, which were otherwise highly regular. Such maxima are typical of samples displaying preferred orientation [Figs. 1 ▸ and 2 ▸(*c*)].

In addition to the azimuthal intensity maxima, some variations in lattice parameters could be observed, probably due to inhomogeneities in mixing and hydration within the large sample volumes (5 × 5 mm). Consistent ensembles were created using a variety of filtering routines (details are given in *Methods* [Sec sec3]).

The magnitude and direction of the orientation effect within each sample chamber were determined automatically and mapped as shown in Figs. 2 ▸(*c*)–2(*e*). The grouping of consistent pixels in the composite map [Fig. 2 ▸(*f*)] indicates the presence of micrometre-scale domains exhibiting preferred orientation. A total of four temperature points were studied along the 50% CTAB–H_2_O isopleth, resulting in ∼3800 SAXS patterns with consistent phase and lattice parameters, exhibiting no intensity fluctuations beyond the observed preferred orientation. These four ensembles were the focus of subsequent PADF analysis in which we explored how this preferred-orientation distribution is manifested.

### 4.2. Pair-angle distribution function analysis

**Figure 3:**
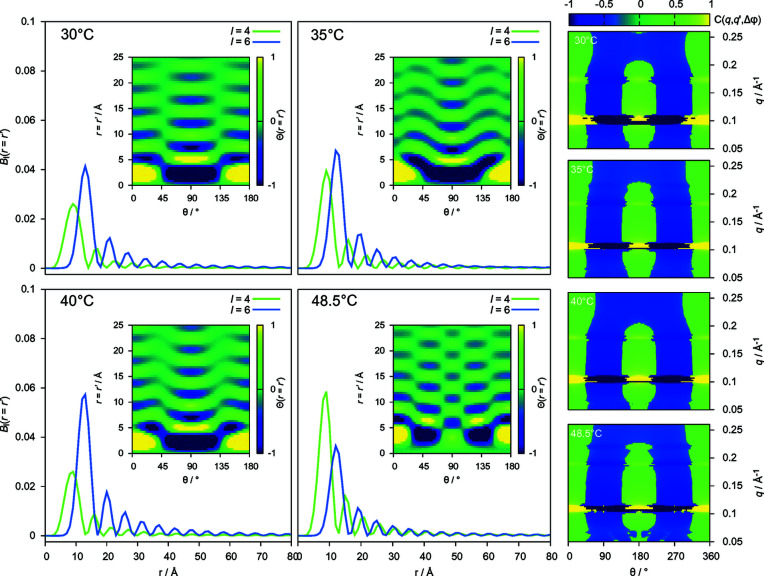
Temperature effects on real-space spherical harmonic coefficients. 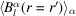 and Θ(*r* = *r*′, θ) slices (insets) for CTAB–H_2_O (50 mol%) mesophases at increasing temperature. The right-hand images show corresponding *q*-space *q* = *q*′ correlation slices for the corresponding temperatures denoted on the image.

PADFs were calculated for CTAB–H_2_O data at four temperature points. The PADF is a three-dimensional volume, and one intuitive visualization is to consider the slice through this volume with *r* = *r*′ as plotted in the insets of Fig. 3 ▸. In each PADF slice shown, the dominant contribution in all cases is the lowest-order *l* = 2 term as indicated by the strong correlation intensity at angles of 0 and 180°. Fig. 3 ▸ also displays the higher-order 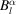 contributions from *l* = 4 and *l* = 6 terms, in all cases normalized to the corresponding *l* = 2 term. Firstly, as expected from the complete absence of nanoscale contributions to the angular intensity variations, the resulting angles and distances of high correlation are incompatible with the known geometry of the hexagonal mesophase. Secondly, the PADF is highly sensitive to subtle shifts in the relative contributions of higher-order terms, even in the presence of a large dominating contribution (*l* = 2). Consider the visual change in Θ(*r* = *r*′) on heating from 30 to 35°C, the *l* = 4 term increases by *ca.* 2%; however, this is sufficient to contribute distinct additional intensity to the PADF. Intensity from the *l* = 4 term reaches a peak of 6% at 48.5°C, rising above the *l* = 6 term and producing an even more marked contribution of intensity at 45°, and 135° in the Θ(*r* = *r*′) slice.

To visualize changes in the corresponding diffraction-pattern ensembles with temperature we plot the *q* = *q*′ slices through the correlation functions *C*(*q*, *q*′, Δϕ) in Fig. 3 ▸(right). Here we have subtracted the radial (*q*) average to show the weak fluctuations from the mean. As expected from the dominance of the *l* = 2 terms of the PADF, the most notable feature is the twofold intensity modulation peaking at 0°, 180° and 360°. Given this sensitivity, can we systematically deduce what changes are occurring in the ensemble orientation distribution function leading to shifts in 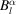 contributions?

### 4.3. Simulated pole distributions

**Figure 4:**
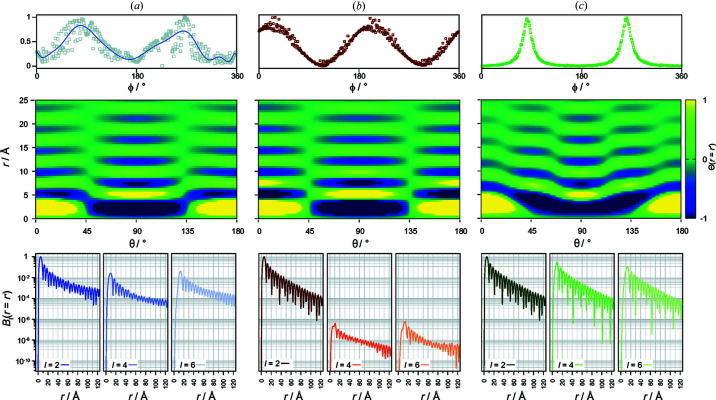
Comparison of pole-distribution effects on real-space spherical harmonic coefficients 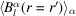 . (*a*) A typical experimentally observed pole-distribution function (top), resulting Θ(*r* = *r*′) slice (middle) and spherical harmonic coefficients (bottom). The experimental distribution most closely resembles the sinusoidal modulation in (*b*). The more peaked Lorentzian modulation (*c*) produces distinct features clearly shown in both the PADF slices (middle) and the magnitudes of the spherical harmonic coefficients (bottom).

The data in Fig. 3 ▸ are shown again in Fig. 4 ▸(*a*), in this case including the *l* = 2 component. As can be seen, the *l* = 4 and *l* = 6 contributions are very small [less than 6%, as shown in Fig. 3 ▸(*a*)]. To explore how changes in the ensemble orientation distribution function are manifested in the PADF, we created a series of simulated diffraction patterns (see *Methods* [Sec sec3]) exhibiting angular intensity modulations following two schemes. Our first model [Fig. 4 ▸(*b*)] consisted of a sinusoidal modulation with random phase, where the inset shows an example of the intensity profile around the diffraction pattern. As can be seen in the *B*_*l*_(*r* = *r*′) plots, this results in a PADF consisting almost entirely of the *l* = 2 term, while the *l* = 4 and *l* = 6 terms have negligible value. This effectively replicates the PADF observed experimentally at 30°C, whose *B*_*l*_(*r* = *r*′) profile is shown in Fig. 4 ▸(*a*).

We now consider what underlying changes to the pole distribution function could lead to the growth of the higher-order terms at the highest temperature of 48.5°C. Notably, these terms contribute to higher-frequency intensity modulations, which implies that the underlying distribution is becoming more sharply peaked.

A second form of intensity modulation was simulated to replicate this effect. In this case, using a Lorentzian peak shape [Fig. 4 ▸(*c*)]. Examining the resulting Θ(*r* = *r*′) slice from this simulation we observe a significant increase in the magnitude of the higher-order contributions, particularly the *l* = 4 term. This term produces the increased correlation intensity observable at 45 and 135° in the Θ(*r* = *r*′) slice in close agreement with the 48.5°C experimental data (Fig. 3 ▸).

These simulations confirm that microtextural effects can explain all the features of the experimentally derived PADFs obtained using the large SAXS/WAXS beam. Secondly, when operating in the 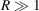 regime, the PADF becomes a highly sensitive probe of the pole distribution, and FXS–PADF experiments could be used to extract microtextural texture information as a by-product of nanostructural studies.

### 4.4. Nanostructure observations

As part of a subsequent study into hexagonal mesophases, we carried out further experiments on CTAB–H_2_O at the XFM beamline at the Australian Synchrotron. The XFM incident beam area is ∼1500 times smaller than that of SAXS/WAXS; therefore, providing the opportunity to test the effect of reduced incident beam size for reducing the relative influence of texture. Although these experiments do not directly compare samples with identical microstructures (compositions differ by 15 mol% CTAB), it does allow us to compare the influence of texture within the sample.

**Figure 5:**
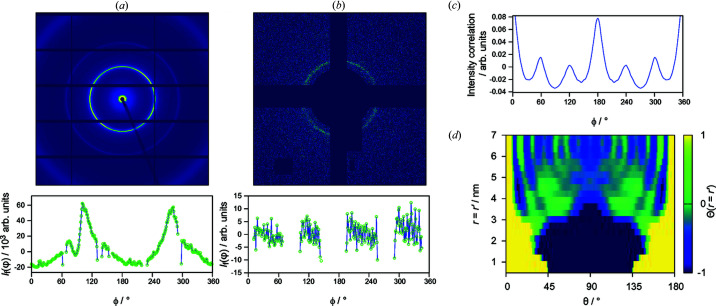
Emergence of nanoscale features. Typical diffraction patterns from CTAB–H_2_O (40 mol% CTAB) hexagonal mesophases data collected with (*a*) a 25 × 250 µm SAXS beam and (*b*) a 4 × 4 µm XFM beam. Profiles indicate the intensity fluctuations (*i.e.* relative to the mean) around the (10) diffraction peak. Omitted regions are covered by detector masks. (*c*) Angular correlations of the (10) diffraction ring from XFM data showing clear peaks at *n*π/3; (*d*) *r* = *r*′ slice through the data collected at the XFM beamline. Nanoscale structural features can be observed below 6 nm.

By comparison with previously studied lipid mesophases (Martin *et al.*, 2020*b* ▸), the scattering from CTAB–H_2_O remains challenging with only weakly anisotropic scattering occurring at low intensities. Critically, however, no clear preferred-orientation effect could be observed in the diffraction patterns, as shown for representative frames in Figs. 5 ▸(*a*) and 5 ▸(*b*). Despite the weak scattering, calculation of the correlation functions for 5000 diffraction patterns reveals the appearance of clear sixfold (60°) symmetry correlations at the hexagonal (10) peak *q* position [Fig. 5 ▸(*c*)], demonstrating that the number of domains does not dominate the balance of contributions to the PADF in this scattering regime. Once transformed to the PADF, we observe clear angular structure, in marked contrast to the SAXS/WAXS data dominated by preferred orientation [Fig. 4 ▸(*a*)].

The nanostructural features of this and additional data in the composition series is beyond the scope of the present article but will be investigated as part of future work. However, some immediate comparisons can be made with previous PADF studies of hexagonal mesophases formed by monoolein buffer mixtures (Martin *et al.*, 2020*b* ▸) conducted on the SAXS/WAXS beamline. In the current study, we only observed structure up to *r* = 6 nm, *i.e.* within the unit cell, while in the work of Martin *et al.* (2020*b* ▸), diffraction patterns were distinctly stronger, and as a result, the PADF showed structure out to a limit of *r* = 25 nm revealing correlations between the hexagonal channels of the mesophase characterized by strong correlation peaks at 60°. This demonstrates that a balance must be struck between attaining strong scattering to high *q* and avoiding contamination from microtexture contributions.

## 5. Discussion

The assumption of a uniform random distribution of scatterers is common to many FXS methods, including PADF analysis. It is important for the feasibility of these techniques to identify when this assumption breaks down. Our key theoretical result here is that the preferred-orientation effect scales quadratically, *i.e.* with the square of the number of illuminated domains ( 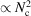 ), while the desired FXS nanostructure term scales linearly (∝ *N*_c_). Hence, even if the orientation preference is a slight deviation from uniformity, it can dominate when the number of domains becomes very large. We present a clear experimental example of precisely this case where the size of the orientation anisotropy is small, as shown by the very smooth diffraction rings. The observed residual anisotropy has the smooth sinusoidal profile expected from texture simulations both for the diffraction patterns and the PADF plots. We did not observe the expected nanostructure correlations in *q* space or the PADF plots for this large-beam dataset, consistent with a large number of domains per exposure, causing the texture-correlation terms to dominate the nanostructure terms. However, by repeating our experiment using a far smaller beam, we observed intensity correlations in *q* space and in the PADF derived from the underlying nanostructure. This extends the capability of the FXS technique to materials with far smaller domains than those observed in previous work (Martin *et al.*, 2020*b* ▸).

In the case of crystal data, there are qualitative differences in the diffraction signal, and the nanostructure correlations for a crystal are sharp clear peaks (Fig. 1 ▸). The angular peaks in the PADF have locations determined by the lattice parameters. The texture terms, on the other hand, have broad angular correlations in *q* space and produce angular PADF peak locations that cannot be explained by the lattice type. Hence, one could envisage the future development of methods to estimate the relative contributions of texture and nanostructure terms for crystals. For mesoscopic texture without a global preferred-orientation axis, it may even lead to methods to recover the orientation distribution for angular intensity-correlation analysis. This is because mesoscopic texture appears to satisfy the conditions specified by FXS three-dimensional imaging methods.

As discussed above, texture effects can often be a complicating factor in structural analysis (*e.g.* X-ray powder diffraction), and the PADF technique is no different in this regard. In the presence of dominating texture that swamps the nanoscale structure, equation (19) shows that reducing the number of contributing crystals/domains in the incident beam will mitigate the texture contribution *B*_t_. Experimentally, this is not always a trivial change; beam sizes are often fixed, or the initial choice of beam size could become inappropriate if the sample microstructure undergoes significant changes through the experiment.

## 6. Conclusions

Macroscopic sample-specific phenomena such as preferred orientation or strain can have a significant effect on fluctuation-correlation methods. Identifying and correcting for macroscopic sample conditions is essential for the broader application of fluctuation methods to phenomena such as phase transitions in real systems. Here we show that preferred orientation can be easily identified by characteristic changes to *q*-space and real-space correlation plots. These results demonstrate the underlying interrelations between crystal-domain number and microtexture and how this may be circumvented by judicious choice of incident-beam size. 

## References

[bb1] Adams, P., Binns, J., Greaves, T. L. & Martin, A. V. (2020). *Crystals*, **10**, 724.

[bb2] Aghamohammadzadeh, H., Newton, R. H. & Meek, K. M. (2004). *Structure*, **12**, 249–256. 10.1016/j.str.2004.01.002 14962385

[bb3] Ahtee, M., Nurmela, M., Suortti, P. & Järvinen, M. (1989). *J. Appl. Cryst.* **22**, 261–268.

[bb4] Australian Synchrotron (2019). *SAXS Software* – *scatterBrain.* http://archive.synchrotron.org.au/aussyncbeamlines/saxswaxs/software-saxswaxs.

[bb5] Auvray, X., Petipas, C., Anthore, R., Rico, I. & Lattes, A. (1989). *J. Phys. Chem.* **93**, 7458–7464.

[bb6] Bunge, H.-J. (1982). *Texture Analysis in Materials Science.* Oxford: Butterworth-Heinemann.

[bb7] Bunge, H.-J. (1987). *Int. Mater. Rev.* **32**, 265–291.

[bb8] Cervellino, A. & Frison, R. (2020). *Acta Cryst.* A**76**, 302–317. 10.1107/S2053273320002521 PMC7233016 32356781

[bb9] Cho, S. Y., Yun, Y. S., Jang, D., Jeon, J. W., Kim, B. H., Lee, S. & Jin, H. J. (2017). *Nat. Commun.* **8**, 74. 10.1038/s41467-017-00132-3 PMC5509745 28706182

[bb10] Dingley, D. J. & Randle, V. (1992). *J. Mater. Sci.* **27**, 4545–4566.

[bb11] Dippel, A.-C., Roelsgaard, M., Boettger, U., Schneller, T., Gutowski, O. & Ruett, U. (2019). *IUCrJ*, **6**, 290–298. 10.1107/S2052252519000514 PMC6400183 30867926

[bb12] Dollase, W. A. (1986). *J. Appl. Cryst.* **19**, 267–272.

[bb13] Donatelli, J. J., Zwart, P. H. & Sethian, J. A. (2015). *Proc. Natl Acad. Sci. USA*, **112**, 10286–10291. 10.1073/pnas.1513738112 PMC4547282 26240348

[bb14] Engler, O. & Randle, V. (2010). *Introduction to Texture Analysis: Macrotexture, Microtexture, and Orientation Mapping.* 2nd ed. Boca Raton: CRC Press.

[bb15] Fuentes, L. (1998). *Textures and Microstructures*, **30**, 167–189.

[bb16] Harris, C. R., Millman, K. J., van der Walt, S. J., Gommers, R., Virtanen, P., Cournapeau, D., Wieser, E., Taylor, J., Berg, S., Smith, N. J., Kern, R., Picus, M., Hoyer, S., van Kerkwijk, M. H., Brett, M., Haldane, A., del Río, J. F., Wiebe, M., Peterson, P., Gérard-Marchant, P., Sheppard, K., Reddy, T., Weckesser, W., Abbasi, H., Gohlke, C. & Oliphant, T. E. (2020). *Nature*, **585**, 357–362. 10.1038/s41586-020-2649-2 PMC7759461 32939066

[bb17] Hilgenkamp, H. & Mannhart, J. (2002). *Rev. Mod. Phys.* **74**, 485–549.

[bb18] Howard, D. L., de Jonge, M. D., Afshar, N., Ryan, C. G., Kirkham, R., Reinhardt, J., Kewish, C. M., McKinlay, J., Walsh, A., Divitcos, J., Basten, N., Adamson, L., Fiala, T., Sammut, L. & Paterson, D. J. (2020). *J. Synchrotron Rad.* **27**, 1447–1458. 10.1107/S1600577520010152 32876622

[bb19] Järvinen, M. (1993). *J. Appl. Cryst.* **26**, 525–531.

[bb20] Kam, Z. (1977). *Macromolecules*, **10**, 927–934.

[bb21] Kam, Z., Koch, M. H. J. & Bordas, J. (1981). *Proc. Natl Acad. Sci. USA*, **78**, 3559–3562. 10.1073/pnas.78.6.3559 PMC319609 6943555

[bb22] Kelly, S. J., Wells, H. C., Sizeland, K. H., Kirby, N., Edmonds, R. L., Ryan, T., Hawley, A., Mudie, S. & Haverkamp, R. G. (2018). *J. Sci. Food Agric.* **98**, 3524–3531. 10.1002/jsfa.8863 29288543

[bb23] Kirian, R. A. (2012). *J. Phys. B At. Mol. Opt. Phys.* **45**, 223001.

[bb24] Kurta, R. P., Altarelli, M. & Vartanyants, I. A. (2016). *Advances in Chemical Physics*. Vol. 161, pp. 1–39. Hoboken, New Jersey: John Wiley & Sons, Ltd.

[bb25] Kurta, R. P., Donatelli, J. J., Yoon, C. H., Berntsen, P., Bielecki, J., Daurer, B. J., DeMirci, H., Fromme, P., Hantke, M. F., Maia, F. R., Munke, A., Nettelblad, C., Pande, K., Reddy, H. K., Sellberg, J. A., Sierra, R. G., Svenda, M., Van Der Schot, G., Vartanyants, I. A., Williams, G. J., Xavier, P. L., Aquila, A., Zwart, P. H. & Mancuso, A. P. (2017). *Phys. Rev. Lett.* **119**, 158102. 10.1103/PhysRevLett.119.158102 PMC5757528 29077445

[bb26] Kurta, R. P., Ostrovskii, B. I., Singer, A., Gorobtsov, O. Y., Shabalin, A., Dzhigaev, D., Yefanov, O. M., Zozulya, A. V., Sprung, M. & Vartanyants, I. A. (2013). *Phys. Rev. E*, **88**, 044501. 10.1103/PhysRevE.88.044501 24229307

[bb27] Kurta, R. P., Wiegart, L., Fluerasu, A. & Madsen, A. (2019). *IUCrJ*, **6**, 635–648. 10.1107/S2052252519005499 PMC6608627 31316808

[bb28] Lehmkühler, F., Fischer, B., Müller, L., Ruta, B. & Grübel, G. (2016). *J. Appl. Cryst.* **49**, 2046–2052. 10.1107/S1600576716017313 PMC5139993 27980511

[bb29] Liu, H., Poon, B. K., Saldin, D. K., Spence, J. C. H. & Zwart, P. H. (2013). *Acta Cryst.* A**69**, 365–373. 10.1107/S0108767313006016 23778093

[bb30] Martin, A. V. (2017). *IUCrJ*, **4**, 24–36. 10.1107/S2052252516016730 PMC5331463 28250939

[bb31] Martin, A. V., Bøjesen, E. D., Petersen, T. C., Hu, C., Biggs, M. J., Weyland, M. & Liu, A. C. (2020*a*). *Small*, **16**, 2000828. 10.1002/smll.202000828 32383542

[bb32] Martin, A. V., Kozlov, A., Berntsen, P., Roque, F. G., Flueckiger, L., Saha, S., Greaves, T. L., Conn, C. E., Hawley, A. M., Ryan, T. M., Abbey, B. & Darmanin, C. (2020*b*). *Commun. Mater.* **1**, 40.

[bb33] Mendez, D., Lane, T. J., Sung, J., Sellberg, J., Levard, C., Watkins, H., Cohen, A. E., Soltis, M., Sutton, S., Spudich, J., Pande, V., Ratner, D. & Doniach, S. (2014). *Philos. Trans. R. Soc. B*, **369**, 20130315. 10.1098/rstb.2013.0315 PMC4052857 24914148

[bb34] Mendez, D., Watkins, H., Qiao, S., Raines, K. S., Lane, T. J., Schenk, G., Nelson, G., Subramanian, G., Tono, K., Joti, Y., Yabashi, M., Ratner, D. & Doniach, S. (2016). *IUCrJ*, **3**, 420–429. 10.1107/S2052252516013956 PMC5094444 27840681

[bb35] Paakkari, T., Blomberg, M., Serimaa, R. & Järvinen, M. (1988). *J. Appl. Cryst.* **21**, 393–397.

[bb36] Paddison, J. A. M. (2019). *Acta Cryst.* A**75**, 14–24. 10.1107/S2053273318015632 30575580

[bb37] Pande, K., Donatelli, J. J., Malmerberg, E., Foucar, L., Bostedt, C., Schlichting, I. & Zwart, P. H. (2018). *Proc. Natl Acad. Sci. USA*, **115**, 11772–11777. 10.1073/pnas.1812064115 PMC6243272 30373827

[bb38] Pande, K., Schwander, P., Schmidt, M. & Saldin, D. K. (2014). *Philos. Trans. R. Soc. B*, **369**, 20130332. 10.1098/rstb.2013.0332 PMC4052868 24914159

[bb39] Poulsen, H. F., Wert, J. A., Neuefeind, J., Honkimäki, V. & Daymond, M. (2005). *Nat. Mater.* **4**, 33–36.

[bb40] Qian, X., Zhi, J., Chen, L., Zhong, J., Wang, X., Zhang, Y. & Song, S. (2018). *Composites Part A*, **112**, 111–118.

[bb41] Roe, R. J. (1965). *J. Appl. Phys.* **36**, 2024–2031.

[bb42] Saldin, D. K., Poon, H.-C., Schwander, P., Uddin, M. & Schmidt, M. (2011). *Opt. Express*, **19**, 17318–17335. 10.1364/OE.19.017318 21935096

[bb43] Saldin, D. K., Poon, H. C., Shneerson, V. L., Howells, M., Chapman, H. N., Kirian, R. A., Schmidt, K. E. & Spence, J. C. (2010). *Phys. Rev. B*, **81**, 174105.

[bb44] Seddon, J. M. (1990). *Biochim. Biophys. Acta*, **1031**, 1–69. 10.1016/0304-4157(90)90002-t 2407291

[bb45] Su, R., Seu, K. A., Parks, D., Kan, J. J., Fullerton, E. E., Roy, S. & Kevan, S. D. (2011). *Phys. Rev. Lett.* **107**, 257204. 10.1103/PhysRevLett.107.257204 22243108

[bb46] Treacy, M. M., Gibson, J. M., Fan, L., Paterson, D. J. & McNulty, I. (2005). *Rep. Prog. Phys.* **68**, 2899–2944.

[bb47] Von Dreele, R. B. (1997). *J. Appl. Cryst.* **30**, 517–525.

[bb48] Welzel, U., Ligot, J., Lamparter, P., Vermeulen, A. C. & Mittemeijer, E. J. (2005). *J. Appl. Cryst.* **38**, 1–29.

[bb49] Wochner, P., Gutt, C., Autenrieth, T., Demmer, T., Bugaev, V., Ortiz, A. D., Duri, A., Zontone, F., Grübel, G. & Dosch, H. (2009). *Proc. Natl Acad. Sci. USA*, **106**, 11511–11514. 10.1073/pnas.0905337106 PMC2703671 20716512

[bb50] Wright, S. I., Nowell, M. M. & Bingert, J. F. (2007). *Metall. Mater. Trans. A*, **38**, 1845–1855.

[bb51] Yang, B. & White, J. W. (2006). *Colloids Surf. A*, **277**, 171–176.

[bb52] Zaluzhnyy, I. A., Kurta, R. P., Mukharamova, N., Kim, Y. Y., Khubbutdinov, R. M., Dzhigaev, D., Lebedev, V. V., Pikina, E. S., Kats, E. I., Clark, N. A., Sprung, M., Ostrovskii, B. I. & Vartanyants, I. A. (2018). *Phys. Rev. E*, **98**, 052703.

[bb53] Zaluzhnyy, I. A., Kurta, R. P., Sulyanova, E. A., Gorobtsov, O. Y., Shabalin, A. G., Zozulya, A. V., Menushenkov, A. P., Sprung, M., Ostrovskii, B. I. & Vartanyants, I. A. (2015). *Phys. Rev. E*, **91**, 042506. 10.1103/PhysRevE.91.042506 25974515

[bb54] Zolotoyabko, E. (2009). *J. Appl. Cryst.* **42**, 513–518.

